# The Medico-Chirurgical Society of Central New York

**Published:** 1898-07

**Authors:** 


### THE MEDICO-CHIRURGICAL SOCIETY OF CENTRAL NEW YORK.

The Medico-Chirurgical Society of Central New York held its fifth annual meeting at Syracuse, N. Y., June 2d, 1898.

A novel feature of the meeting was a paper on homing pigeons by Dr. N. H. Haviland, of Fulton, N. Y. He told of the use of homing pigeons as medical messengers. The doctor has about thirty pigeons that he uses in his practice, and three of these he brought to the meeting. After the morning's session Dr. Haviland liberated three pigeons in front of the court-house. He placed a message in a nickel band about the birds' legs, and then tossed them into the air. The pigeons made several circles of the neighborhood, and when they got their bearings they started in the direction of Fulton, twenty-four miles away. Dr. Haviland said that the birds would reach home in about thirty minutes. The messages contained a request to his valet to note the time the birds reach home, and wire the same back to this city.

This paper is printed in full in this number at page 316.

The meeting was presided over by President Dr. W. E. Denel, of Chittenango. The minutes of the last meeting and the financial statement were read by Dr. E. Elmer Keeler, of this city, Secretary and Treasurer of the Society. Several candidates were admitted to membership.

"Acute Inflammatory Diseases of the Lungs in Children" was the first paper read by Dr. F. F. Williams, of Canton. He said that it was rare to find severe bronchitis without pneumonia, and vice versa. A discussion followed.

Dr. A. G. Anthony, of the city, read an interesting paper on "Neuroses of Infancy." Dr. Keeler discussed the paper. Dr. L. A. Martin, of Binghamton, related operative cases of appendicitis, and some clinical cases were reviewed by Dr. W. L. Hartman, of this city.

"Alcohol in Medicine" was the title of the paper read by Dr. E. L. Hinman, of Oswego.

President Denel's annual address was filled with interesting statistics.

After the election of officers this programme was carried out:

Hospital report by J. W. Sheldon, M. D., Syracuse, President of the staff. "One Case in Which the Remedy Indicated the Disease Miasm Before It Was Known," S. L. Guild-Leggett, M. D., Syracuse; discussion opened by Gordon W. Hoyt, M. D., Syracuse. "Uricacidæmia," C. E. Hinman, M. D., Syracuse; discussion opened by W. C. Du Bois, M. D. "Uric Acid Diathesis," A. F. Mills, M. D., Binghamton. "Typhoid Fever," E. A. Simonds, M. D., Carthage. "Typhoid Fever," comparative statistics, C. D. Hale, M. D., Syracuse. Subject not given, E. B. Nash, Cortland. "Why I Am and What I Am," William M. Gwynn, M. D., Auburn. "The Necessity of the Microscope in Medical and Surgical Diagnosis," R. W. Craffee, M. D., Syracuse. "Diseases of the Labyrinth," H. A. Church, M. D., Syracuse. "Case," J. T. Wallace, M. D., Oneida. "History of a Scarlet Fever Epidemic," A. H. Bruce, M. D., Wolcott. Clinical Cases. Miscellaneous business.

